# Supplementary material for: Naming Game on Networks: Let Everyone be Both Speaker and Hearer
Source: Sci Rep. 2014 Aug 21;4:6149. doi: 10.1038/srep06149 (PMC4139946; doi:10.1038/srep06149)
Supplement: Supplementary Information [file srep06149-s1.pdf]

Supplementary material for the paper:

Naming Game on Networks:
Let Everyone be Both Speaker and Hearer

Yuan Gao, Guanrong Chen and Rosa H. M. Chan

July 25, 2014

The small-world network by WS model with different initial connected neighbour K are investigated, the configuration properties are illustrated in Table S1.

Network	#Nodes	$< D >$	$< PL >$	$< CC >$
SW with $K = 20$ and $RP = 0.2$ ($WS - 20 - 0.2$)	1000	40.0	2.4651	0.3837
SW with $K = 30$ and $RP = 0.2$ ($WS - 30 - 0.2$)	1000	60.0	2.1621	0.3937
SW with $K = 50$ and $RP = 0.2$ ($WS - 50 - 0.2$)	1000	100.0	1.9140	0.4055

Table S1: Small-world Networks with differnt initial connected neighbour K. Where RP is the rewiring probability, $< D >$ is average degree, $< PL >$ is average path length and $< CC >$ is average clustering coefficient.

The Success Ratio and the three metrics for various β on WS networks with different $< D >$ and same $N = 20$ are shown in Figure S1 and Figure S2, respecitively. From them, it can be observed that (i) both N_{total_max} and N_{diff_max} increases with $< D >$, because the Success Ratio of mid-iteration is relatively smaller for networks with larger $< D >$. (ii) N_{iter_cvg} decreases with $< D >$ increasing, the reason is when $< D >$ is small, each node in WS networks only connect with only a few number of its "nearest neighbour", i.e., the whole network are more "locally connected" and the intra-group consensus words are hard to be spread to far away, thus finally leads to a longer convergence time.

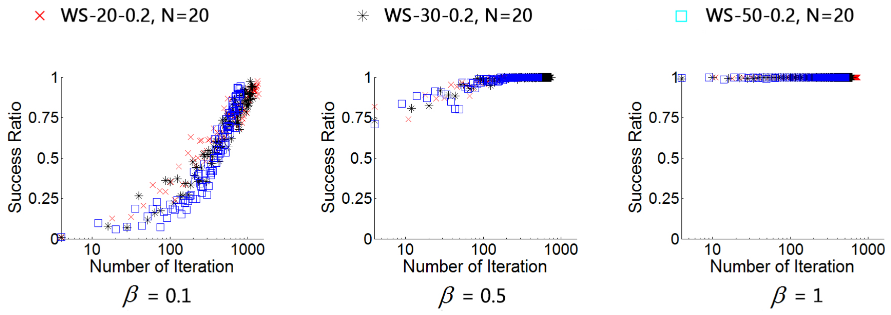

Figure S1: Success Ratio for each iteration with β equal to 0.1, 0.5, and 1 on WS networks with various $< D >$. The group size N for all simulations are 20. The network configurations (in order of RED, BLACK, GLUE) are $WS - 20 - 0.2$, $WS - 30 - 0.2$, $WS - 50 - 0.2$. Columns from left to right are the Success Ratio for β =0.1, 0.5 and 1, respectively.

The comparison of NGG and NGMH with different group size N equal to 10, 50, 100 can be found in Figure S3 - S5, respectively. The analysis of these results is consensus with that in the main text.

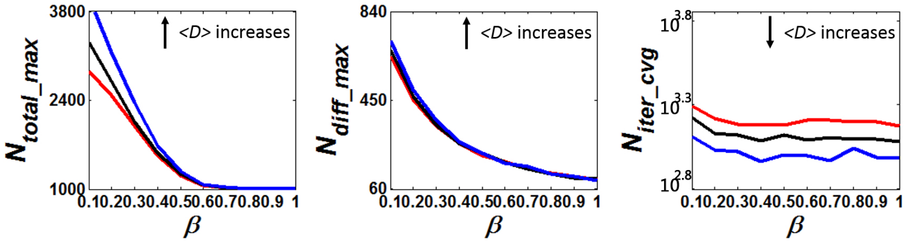

Figure S2: The three convergence metrics for group size $N = 20$ on WS networks with various $< D >$. The network configurations (in order of RED, BLACK, GLUE) are $WS-20-0.2$, $WS-30-0.2$, $WS-50-0.2$. Columns from left to right indicates the illustrations for N_{total_max}, N_{diff_max} and N_{iter_cvg} (log-scale in Y-axis), respectively.

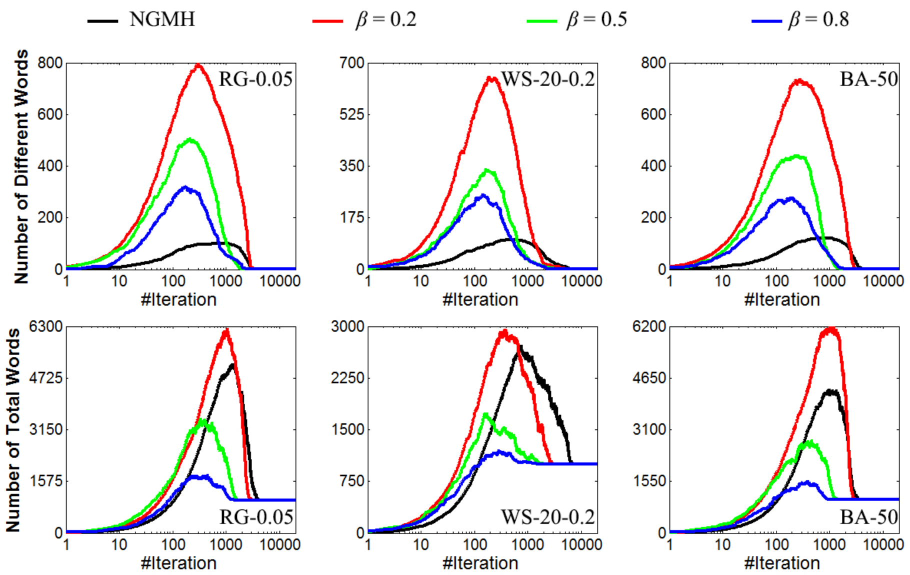

Figure S3: The convergence of the NGG model for group size N equal to 10. Both Number of Different Words vs. #Iteration and Number of Total Words vs. #Iteration are investigated. Three different β as well as NGMH are compared. The tested network configurations are $RG-0.05$, $WS-20-0.2$ and $BA-50$.

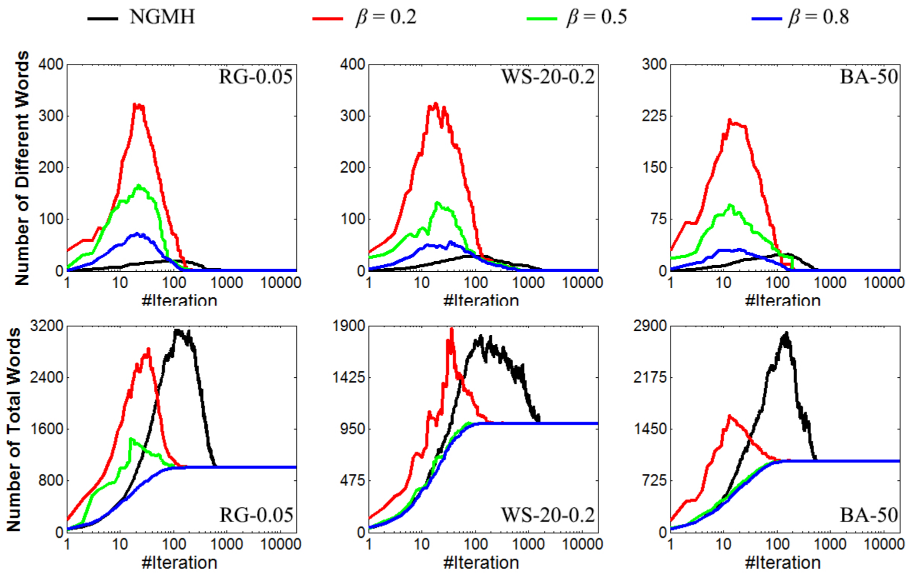

Figure S4: The convergence of the NGG model for group size N equal to 50. Other parameters are same as Figure S3.

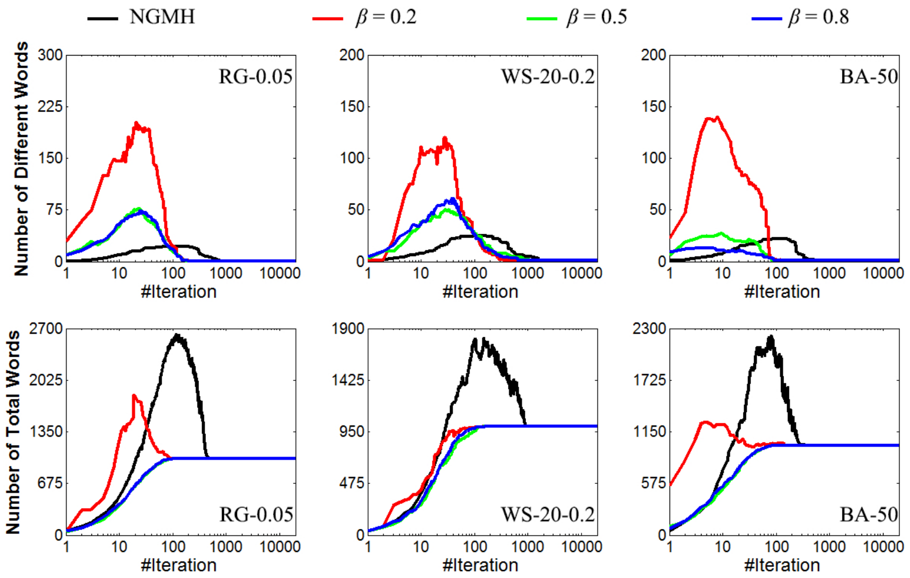

Figure S5: The convergence of the NGG model for group size N equal to 100. Other parameters are same as Figure S3.

The Success Ratio for various β from 0.1 to 0.9 on the network configurations of Table 1 are illustrated in Figure S6 - S8, for RG, WS, BA model respectively. The Success Ratio for all networks types increase monotonously with β

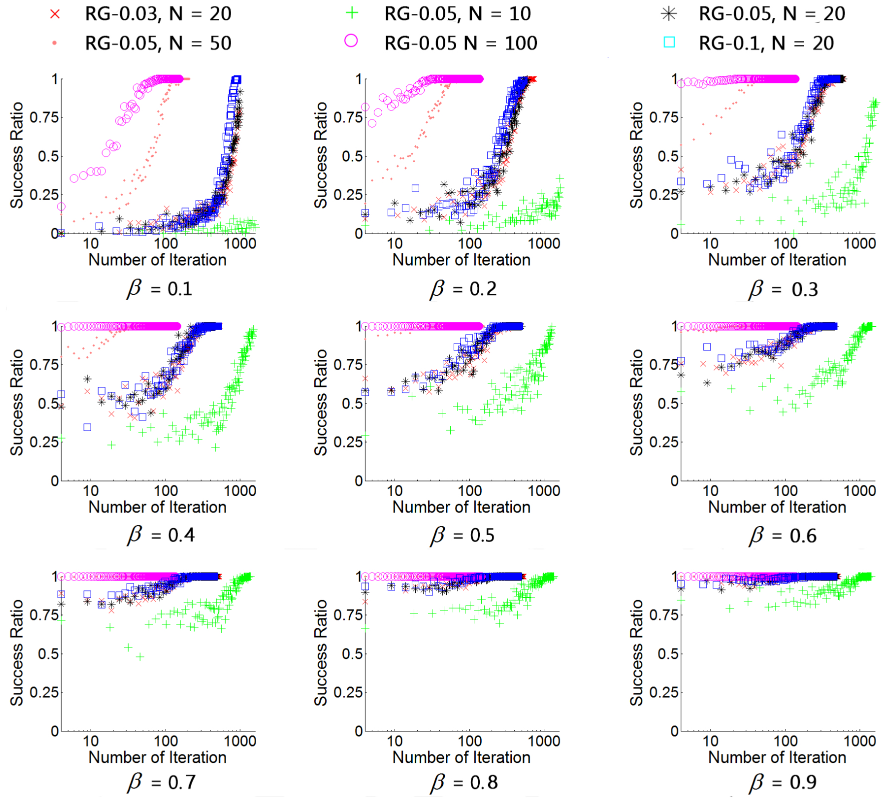

Figure S6: The Success Ratio for various β from 0.1 to 0.9 on the RG networks with various group size N. The tested samples are $RG-0.03$ with $N = 20$, $RG-0.05$ with $N = \{10, 20, 50, 100\}$, $RG-0.1$ with $N = 20$.

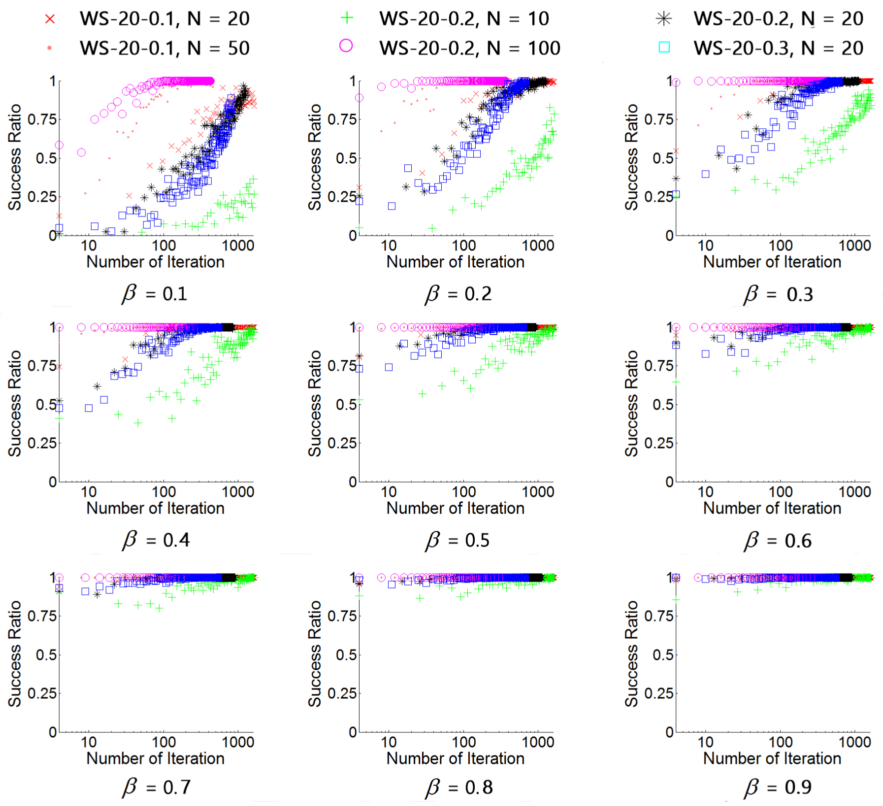

Figure S7: The Success Ratio for various β from 0.1 to 0.9 on the WS networks with various group size N. The tested samples are $WS-20-0.1$ with $N = 20$, $WS-20-0.2$ with $N = \{10, 20, 50, 100\}$, $WS-20-0.3$ with $N = 20$.

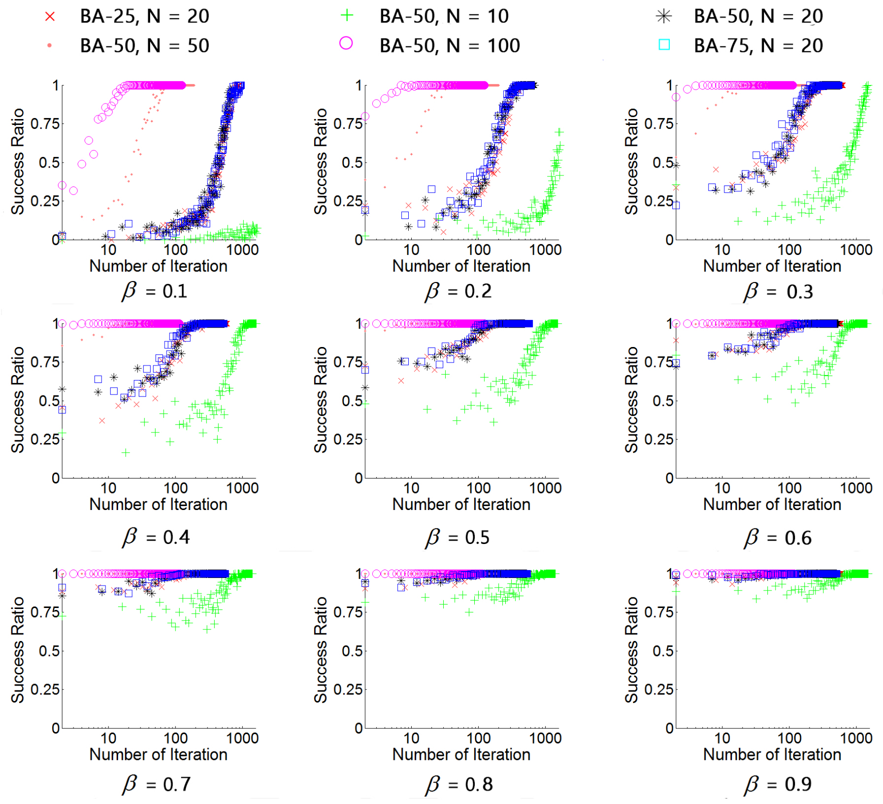

Figure S8: The Success Ratio for various β from 0.1 to 0.9 on the WS networks with various group size N. The tested samples are $BA-25$ with $N = 20$, $BA-50$ with $N = \{10, 20, 50, 100\}$, $BA-75$ with $N = 20$.

For the comparison of three metrics $\mathcal{M}$ (i.e., N_{total_max}, N_{diff_max}, N_{iter_cvg}) between NGMH and our method, the results of NGMH for the network configurations used in main text are listed in Table S2. All observations of Subsection *Convergence of NGG* hold when compare Table S2 with Figure 4 - 6.

Network Configurations	N_{total_max}	N_{diff_max}	N_{iter_cvg}
$RG - 0.03$, $N = 20$	3446.35	50.90	2177.65 ($\approx 10^{3.34}$)
$RG - 0.05$, $N = 10$	4944.00	101.95	4809.15 ($\approx 10^{3.68}$)
$RG - 0.05$, $N = 20$	3722.50	49.95	2080.85 ($\approx 10^{3.32}$)
$RG - 0.05$, $N = 50$	2706.75	21.80	633.95 ($\approx 10^{2.80}$)
$RG - 0.05$, $N = 100$	2629.55	20.15	619.05 ($\approx 10^{2.79}$)
$RG - 0.1$, $N = 20$	3793.75	49.55	1843.25 ($\approx 10^{3.27}$)
$SW - 20 - 0.1$, $N = 20$	1763.35	49.10	8107.75 ($\approx 10^{3.91}$)
$SW - 20 - 0.2$, $N = 10$	2799.70	100.50	8522.20 ($\approx 10^{3.93}$)
$SW - 20 - 0.2$, $N = 20$	2214.35	49.95	4612.85 ($\approx 10^{3.66}$)
$SW - 20 - 0.2$, $N = 50$	1789.65	23.85	1889.50 ($\approx 10^{3.28}$)
$SW - 20 - 0.2$, $N = 100$	1832.45	25.15	1768.20 ($\approx 10^{3.25}$)
$SW - 20 - 0.3$, $N = 20$	2629.85	51.35	3022.90 ($\approx 10^{3.48}$)
$BA - 25$, $N = 20$	3584.30	63.35	2517.50 ($\approx 10^{3.40}$)
$BA - 50$, $N = 10$	5069.85	119.70	6002.25 ($\approx 10^{3.78}$)
$BA - 50$, $N = 20$	3770.70	60.85	2463.50 ($\approx 10^{3.39}$)
$BA - 50$, $N = 50$	2904.60	26.20	771.20 ($\approx 10^{2.89}$)
$BA - 50$, $N = 100$	2235.55	17.15	410.55 ($\approx 10^{2.61}$)
$BA - 75$, $N = 20$	3879.00	59.20	2452.30 ($\approx 10^{3.39}$)

Table S2: The three metrics, N_{total_max}, N_{diff_max} and N_{iter_cvg} on the selected network configurations. The selected network configurations are same as these used in Figure 4 - 6, i.e., $RG - 0.03$ with $N = 20$, $RG - 0.05$ with $N = \{10, 20, 50, 100\}$, $RG - 0.1$ with $N = 20$ for RG networks; $WS - 20 - 0.1$ with $N = 20$, $WS - 20 - 0.2$ with $N = \{10, 20, 50, 100\}$, $WS - 20 - 0.3$ with $N = 20$ for WS networks; and $BA - 25$ with $N = 20$, $BA - 50$ with $N = \{10, 20, 50, 100\}$, $BA - 75$ with $N = 20$ for BA networks.

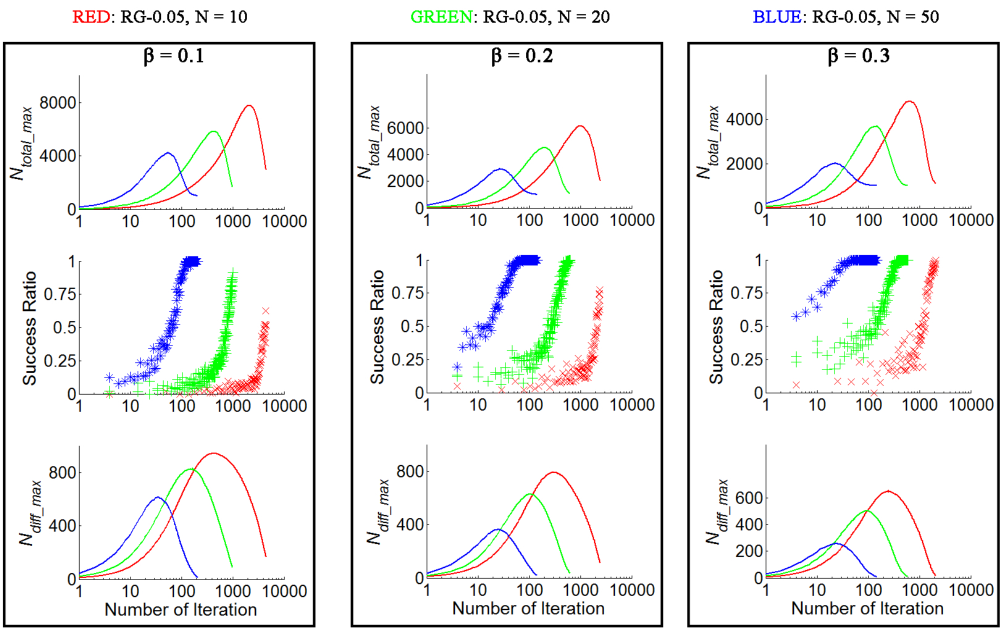

Figure S9: The numerical evidence for the analysis of the alteration of N_{total_max} and N_{diff_max} on RG networks. $RG - 0.05$ with N = 10, 20, 50 are chosen for illustration since the difference of SR among them are significant. β from 0.1 - 0.3 are used, because it is not too large to lead the saturation of SR in the initial iterations. Rows from top to bottom: the N_{total_max}, the SR and the N_{diff_max} of each iterations. Column from left to right: $\beta = 0.1, 0.2, 0.3$.

Figure S9 - S11 provides the numerial evidence of RG, SW, BA networks, respectively, for the analysis of the alteration of N_{total_max} and N_{diff_max}. The increase of N_{total_max} is kept roughly at βN^2 by each iteration without considering the elimation of words, because there is βN words to be transmitted and the group size is N. It is observed in Figure S9 - S11 that both N_{total_max}, N_{diff_max} are achieved at the mid-iterations, higher SR in late iterations does not affect the value of N_{total_max}, N_{diff_max}. And both N_{total_max}, N_{diff_max} are inversely proportional to the SR of mid-iterations.

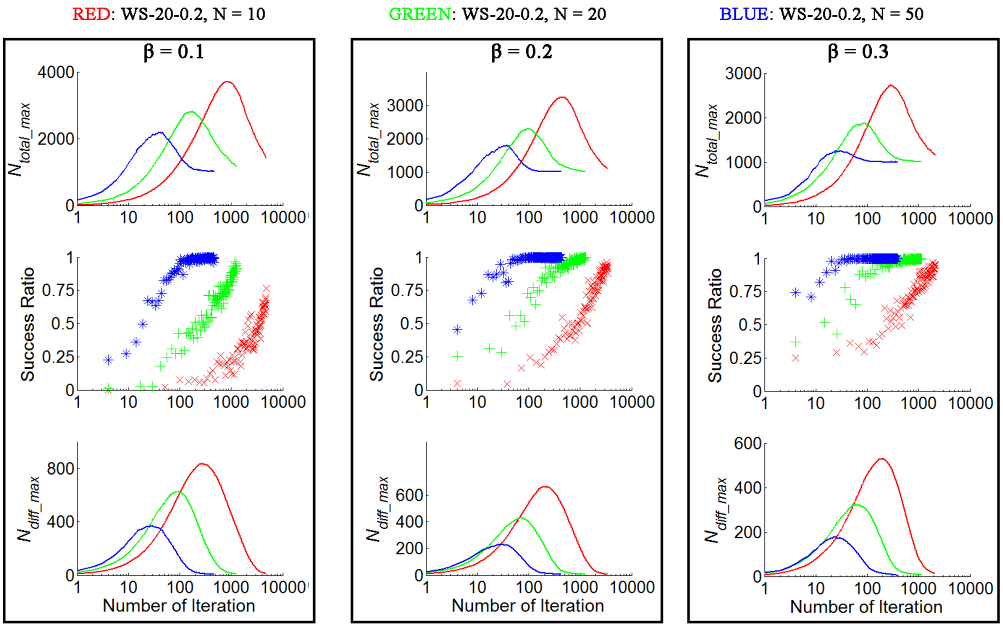

Figure S10: The Success Ratio for various β from 0.1 to 0.9 on the WS networks with various group size N. The tested samples are $BA-25$ with $N = 20$, $BA-50$ with $N = \{10, 20, 50, 100\}$, $BA - 75$ with $N = 20$.

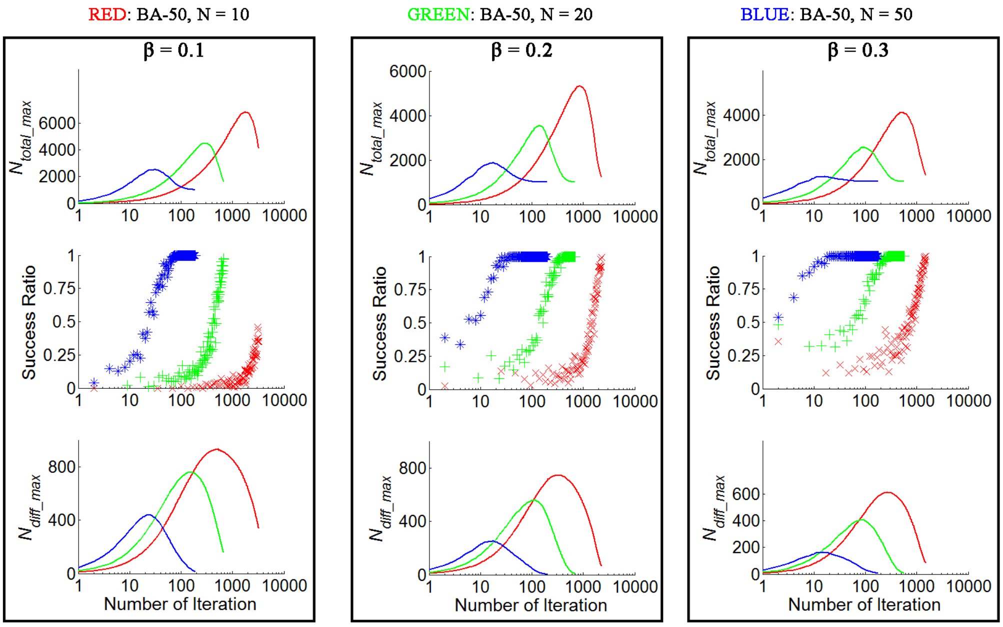

Figure S11: The Success Ratio for various β from 0.1 to 0.9 on the WS networks with various group size N. The tested samples are $BA-25$ with $N = 20$, $BA-50$ with $N = \{10, 20, 50, 100\}$, $BA - 75$ with $N = 20$.
